# Capybara and Brush Cutter Involvement in Q Fever Outbreak in Remote Area of Amazon Rain Forest, French Guiana, 2014

**DOI:** 10.3201/eid2605.190242

**Published:** 2020-05

**Authors:** Jacques-Robert Christen, Sophie Edouard, Thierry Lamour, Enguerrane Martinez, Claire Rousseau, Franck de Laval, François Catzeflis, Félix Djossou, Didier Raoult, Vincent Pommier de Santi, Loïc Epelboin

**Affiliations:** Laveran Military Teaching Hospital, Marseille, France (J.-R. Christen);; Aix-Marseille University, Institut de Recherche pour le Développement, IHU-Méditerranée Infection, Microbes, Evolution, Phylogénie et Infection, Marseille (S. Edouard, D. Raoult);; French Armed Forces Medical Health Service, Cayenne, French Guiana (T. Lamour, E. Martinez);; Clermont-Tonnerre Military Teaching Hospital, Brest, France (C. Rousseau);; French Armed Forces Center for Epidemiology and Public Health, Marseille (F. de Laval, V. Pommier de Santi);; Institute of Evolutionary Sciences, UMR 5554 CNRS, University of Montpellier, Montpellier, France (F. Catzeflis);; Andrée Rosemon Hospital and Amazonian Ecosystems and Tropical Pathology, EA 3593, University of French Guiana, Cayenne (F. Djossou, L. Epelboin);; Aix-Marseille University, Institut de Recherche pour le Développement, Assistance Publique-Hôpitaux de Marseille, French Military Health Service, Vecteurs-Infections TROpicales et MEditerranéennes, IHU-Méditerranée Infection, Marseille (V. Pommier de Santi)

**Keywords:** Q fever, *Coxiella burnetii*, capybara, *Hydrochoerus hydrochaeris*, infectious aerosols, brush cutter, weed cutter, grass trimmer, outbreak, Amazon Rain Forest, French Guiana, Comté River, military personnel, bacteria, zoonoses, pneumonia, multispacer sequence type 17, MST17, sylvatic cycle

## Abstract

We investigated a Q fever outbreak that occurred in an isolated area of the Amazon Rain Forest in French Guiana in 2014. Capybara fecal samples were positive for *Coxiella burnetii* DNA. Being near brush cutters in use was associated with disease development. Capybaras are a putative reservoir for *C. burnetii*.

Q fever is a cosmopolitan zoonosis caused by *Coxiella burnetii*, a gram-negative coccobacillus. Transmission occurs mainly through inhalation of contaminated particles present in the environment. Cattle, sheep, and goats constitute the main reservoirs worldwide, and afterbirth from infected animals is highly contagious.

The incidence of Q fever is particularly high in French Guiana, an overseas entity of France located in the Amazon region of South America between Suriname and the state of Amapá in Brazil. Disease in French Guiana is caused by a unique genotype, *C*.* burnetii* multispacer sequence type 17 (MST17) (*1*–*3*). The disparities in incidence between French Guiana and its neighboring countries suggest that Q fever incidence is underestimated in that part of the world, potentially because of misdiagnosis or the unavailability of diagnostic tools (*4*). Cases occur mainly in Cayenne, the capital of French Guiana, and the surrounding areas (*5*). Outbreaks beyond the outskirts of Cayenne have not been described. Studies of multiple domestic and wild fauna in French Guiana have only revealed the 3-toed sloth (*Bradypus tridactylus*) as a potential *C*. *burnetii* reservoir (*6*–*9*). However, another wild animal reservoir is highly suspected for this bacterium (*4*), and the epidemiologic cycle in French Guiana remains incomplete. In this article, we describe an outbreak that occurred in 2014 among French Navy service members in a remote area of French Guiana and the outbreak investigation findings, which implicated another species as a potential *C*. *burnetii* reservoir.

## The Study

During August–September 2014, a total of 5 Q fever cases were diagnosed among 12 French Navy service members. All had been deployed for 3 days in mid-August to a carbet (an open-sided wooden shelter surrounded by forest) located on the Comté River, 30 km south of Cayenne (4°37′57.44′′N, 52°23′49.5′′W). Q fever diagnostic tests (Q Fever IFA IgM and Q Fever IFA IgG; Focus Diagnostics, http://www.focusdx.com; 100% sensitivity and 99% specificity to *C. burnetii* Nine Mile strain) were performed at the Institut Pasteur in Cayenne.

Patient interviews revealed that they had spent 3 days cleaning the carbet with brooms and clearing the surrounding area using a brush cutter (also known as a weed cutter or grass trimmer). No other common exposure was found. We performed a retrospective case–control study that included the 5 cases and 15 controls. Control participants had spent multiple days at the carbet during the 3-day cleanup (7 controls) or during the weeks just before or after the cleanup (8 controls) without developing any symptoms. We also administered a questionnaire to assess activities potentially associated with the *C. burnetti* exposure.

**Figure 1:**
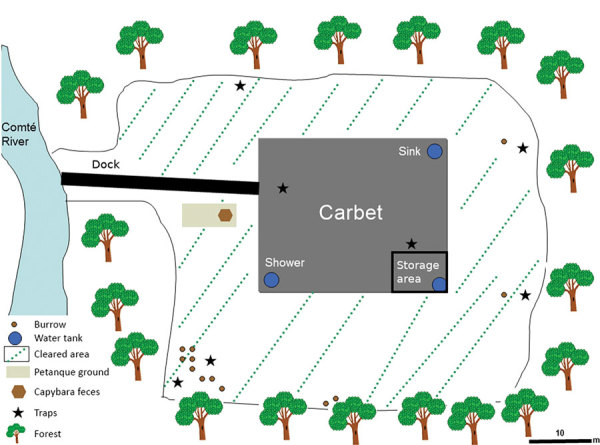
Sample collection and animal trap sites around carbet used in environmental investigation of Q fever outbreak near Comté River in the Amazon Rain Forest area of French Guiana, 2014.

Given the unexpected location of this outbreak (i.e., in an isolated spot in the middle of the rain forest, far from Cayenne), we performed an environmental investigation for *C. burnetti *in late September. We sampled several possible sources in and around the carbet: dust from the storage area; soil from under the carbet; soil from burrows of small, nonflying mammals; mammal feces and bird and reptile droppings; and water from the sink, shower, shower water tank, and toilets (Figure 1). To sample small mammals around the carbet, we used 12 animal traps from BTT Mécanique (https://www.bttmecanique.fr), Tomahawk Live Trap (https://www.livetrap.com), and H.B. Sherman Traps, Inc. (https://www.shermantraps.com) per night for 5 nights. With all samples, we performed a quantitative real-time PCR (qPCR) targeting IS*1111* as previously described (*10*). We confirmed all positive results (i.e., DNA samples with a cycle threshold <35) using a second qPCR targeting the IS*30a *repeat sequence. We used another qPCR specific for *C. burnetii* MST17 to genotype *C. burnetii* DNA–positive samples (*11*).

We confirmed the 5 cases of acute Q fever (in 4 men and 1 woman of median age 27 [range 20–40] years) by serology (Table 1); the attack rate was 40% (5/12). Symptoms began 12–23 days after the stay; 4 patients had elevated fever (>39°C) and pneumonia with >1 lobe involved (Table 1). Each Q fever patient received 200 mg of doxycycline daily for 3 weeks. Outcomes were favorable, and none progressed to persistent focalized Q fever. The only risk factor found in univariate analysis was being close to the brush cutter during brush cutting (p = 0.03; Table 2).

**Table 1:** Characteristics of French Navy service members who had Q fever after visit to carbet along Comté River, Amazon Rain Forest, French Guiana, 2014–2015*

Patient no.	Age, y/sex	Dates of stay at carbet	Date of symptom onset	Date of serology	Phase I			Phase II		Clinical presentation
					IgG	IgM		IgG	IgM	
1	21/M	2014 Aug 15–17	2014 Sep 2	2014 Sep 9	<50	800		50	100	Fever, pneumonia, myalgia
				2014 Sep 15	<50	>6,400		100	6,400	
2	40/F	2014 Aug 15–17	2014 Aug 29	2014 Sep 1	<50	<50		<50	<50	Fever, pneumonia
				2014 Sep 10	<50	400		3,200	6,400	
3	27/M	2014 Aug 15–17	2014 Aug 31	2014 Sep 5	<50	<50		<50	50	Fever, pneumonia, myalgia
				2014 Sep 17	<50	6,400		800	>6,400	
4	20/M	2014 Aug 15–17	2014 Aug 31	2014 Sep 26	100	800		200	800	Fever, myalgia
5	28/M	2014 Aug 15–17	2014 Sep 13	2014 Sep 18	<50	<50		<50	<50	Fever, pneumonia, vomiting, diarrhea, myalgia
6	29/F	2014 Dec 22	2015 Jan 14	2015 Jan 28	200	800		800	200	Fever, pneumonia
				2015 Feb 24	50	1,600		800	800	

*We measured antibody concentrations against both antigenic variants (phase I and phase II) of* Coxiella burnetii*. High levels of phase II antibodies are found in acute Q fever, whereas high levels of IgG phase I antibodies are predominant in chronic Q fever.

**Table 2:** Univariate analysis of risk factors for acute Q fever development in outbreak near Comté River, Amazon Rain Forest, French Guiana, 2014*

Exposure type	Cases, n = 5, no. (%)	Controls, n = 15, no. (%)	Crude OR (95% CI)	p value
Using brush cutter	2 (40)	3 (20)	2.52 (0.15–36.3)	0.56
Being close to brush cutter while in use	5 (100)	5 (33)	NA (1.20–NA)	0.03
Collecting or moving wood	4 (80)	11 (73)	1.43 (0.09–89.2)	1
Collecting freshly cut grass	0	2 (13)	0 (0–16.8)	1
Cleaning dust on furniture	2 (40)	11 (73)	0.26 (0.02–3.19)	0.29
Sweeping carbet	3 (60)	13 (87)	0.25 (0.01–4.79)	0.25
Using shower inside carbet	3 (60)	4 (27)	3.81 (0.31–62.5)	0.29
Walking around carbet	2 (40)	5 (33)	1.31 (0.08–16.0)	1
Stirring soil	1 (20)	1 (7)	3.24 (0.04–293)	0.45
Cleaning animal droppings	0	7 (47)	0 (0–1.81)	0.11

*NA, not applicable; OR, odds ratio.

**Figure 2:**
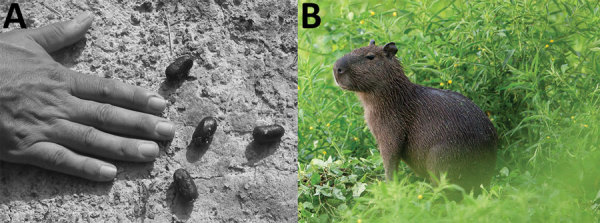
Feces of capybara (*Hydrochoerus hydrochaeris*) (A) and image of capybara (B), French Guiana. The length of the middle fingernail, which is often used in the field for feces measurement, is 12 mm. Photographs by Nicolas Defaux, http://www.photographienature.com.

We failed to capture any small mammals during the 5-day sampling period. All qPCR tests performed with dust, soil samples, water samples, bird and reptile droppings, and fecal samples taken from burrows (attributable to small mammals) were negative for *C. burnetti* DNA. However, fresh mammal fecal samples were positive for *C. burnetii* (cycle threshold 31) and later genotyped as MST17. These fecal samples were identified by 3 independent experts as originating from capybaras (*Hydrochoerus hydrochaeris*, the world’s largest rodent; Figure 2), which were often found laying a few meters from the carbet. We did not perform DNA testing to confirm species origin, but capybara feces are distinctive and easily identified by their descriptions in the literature (*12*,*13*).

To prevent further Q fever cases, the carbet was closed for 4 months. In late December 2014, eight service members were sent to clean up the grounds surrounding the carbet. Instructions were issued to wear personal protective equipment (coveralls, gloves, and an FFP [filtering face piece] 2) during the operation. Only 1 young woman did not comply with the instructions for wearing the mask because she did not use the brush cutter herself; 3 weeks later, she had Q fever pneumonia (Table 1). None of the other service members got sick.

## Conclusions

In total, 5 of 6 Q fever patients had pneumonia, confirming the virulence of MST17, the sole genotype found in French Guiana (*3*). This Q fever outbreak occurred outside of Cayenne and suburban areas, in the Amazon Rain Forest. These results confirm wildlife exposure and a sylvatic transmission cycle for *C. burnetii* MST17. In this outbreak, the capybara appeared to excrete *C. burnetii* in its feces, which caused environmental contamination that persisted for several months; alternatively, the environment might have still been infectious months later because of recontamination. Other wild animals in the Amazon Rain Forest might also be able to excrete *C. burnetii *(*14*,*15*), so the sites of future outbreaks cannot be predicted. The hazard of a *C. burnetii *infection probably exists throughout the entire rain forest because no barriers limit animal mobility in this environment.

Overlap between the sylvatic cycle and risky human activities led to this Q fever outbreak, and the availability of diagnostic tools led to its detection. Diagnostic tool availability could explain the lack of detection elsewhere in the Amazon region. Our case–control study found that being close to the brush cutter during its operation was associated with disease development. The unfortunate incident responsible for the sixth case 4 months after the initial outbreak confirmed this association; this incident demonstrated that the brush cutter, a tool commonly used for gardening in French Guiana, generated an infectious aerosol when used to cut vegetation near contaminated soil. This incident also demonstrated that wearing an FFP2 is effective protection against infection in cases of exposure to *C. burnetii* infectious aerosols*.* Outbreaks occur when human activities lead to aerosolization of elements in the environment contaminated during the sylvatic cycle. As shown by this report, we cannot control sylvatic cycle transmission, but protective measures implemented during risky activities can prevent infections in humans.
